# Advances in Cardiac Imaging and Genetic Testing for Diagnosis and Risk Stratification in Cardiomyopathies: 2024 Update

**DOI:** 10.3390/jcm13237166

**Published:** 2024-11-26

**Authors:** Tomasz Gasior

**Affiliations:** Collegium Medicum—Faculty of Medicine, WSB University, 41-300 Dabrowa Gornicza, Poland; tgasior@wsb.edu.pl

**Keywords:** cardiomyopathy, genetic testing, cardiac imaging, echocardiography, HCM, DCM, ACM, ARVC, artificial intelligence

## Abstract

Cardiomyopathies represent a diverse group of heart muscle diseases marked by structural and functional abnormalities that are not primarily caused by coronary artery disease. Recent advances in non-invasive imaging techniques, such as echocardiography, cardiac magnetic resonance, and computed tomography, have transformed diagnostic accuracy and risk stratification, reemphasizing the role of cardiac imaging in diagnosis, phenotyping, and management of these conditions. Genetic testing complements imaging by clarifying inheritance patterns, assessing sudden cardiac death risk, and informing therapeutic choices. Integrating imaging data, such as left ventricular wall thickness, fibrosis, and apical aneurysms, with genetic findings enhances decision-making for implantable cardioverter-defibrillators in high-risk patients. Emerging technologies like artificial intelligence, strain imaging, and molecular imaging, alongside genetic testing, hold the promise of further refining diagnosis and personalized treatment approaches. This article summarizes the current state and future perspectives of cardiac imaging and genetic testing for diagnosis and risk stratification in cardiomyopathies, offering practical insights for patients’ management.

## 1. Introduction

Cardiomyopathies are a heterogeneous group of diseases characterized by structural and functional abnormalities in the myocardium and represent a high unmet medical need and global burden being a frequent cause of heart failure or sudden death, particularly in young patients. Since their original description, major advances have been made in the phenotyping and natural course of the disease leading to different classification systems. The most recent European Society of Cardiology (ESC) guidelines describe a variety of phenotypes such as hypertrophic cardiomyopathy (HCM), dilated cardiomyopathy (DCM), non-dilated left ventricular cardiomyopathy (NDLVC), arrhythmogenic right ventricular cardiomyopathy (ARVC), and restrictive cardiomyopathy (RCM) [1]. The classification of cardiomyopathy proposed by the ESC is based on the phenotype, although an integrated etiological diagnosis should also take into account other features such as the presence of arrhythmias and/or AV conduction disorders, analysis of the patient’s pedigree, involvement of other organs, analysis of laboratory biomarkers, and results of histopathological and genetic tests. Despite the importance of phenotype in the diagnosis, it is important to recognize the potential for phenotype evolution due to the progression of hemodynamic disorders, as well as the influence of modulating factors such as hypertension, diabetes, sports, obesity, toxic factors, or pregnancy on the expression of heart dysfunction, dilatation, hypertrophy, and arrhythmia.

Over the past decade, non-invasive cardiac imaging has transformed the landscape of diagnosis, phenotyping, risk stratification, and management of these disorders. In light of recent advances, two new documents have been published in 2024. The American Heart Association (AHA) and American College of Cardiology (ACC) guidelines have provided updated recommendations emphasizing the critical role of imaging in HCM [2]. This article explores the significant advances in cardiac imaging modalities, including echocardiography, cardiac magnetic resonance (CMR), and computed tomography (CT), which are crucial for the accurate diagnosis, phenotyping, and management of patients with cardiomyopathies. Also in 2024, the Task Force consensus report by D. Corrado et al. was published, proposing new diagnostic criteria and a new classification of arrhythmogenic cardiomyopathy (ACM) [3]. In both documents, experts suggested redefining an arrhythmogenic cardiomyopathy (ACM) phenotype with three subphenotypes: dominant-right phenotype (ARVC), arrhythmogenic biventricular phenotype (ABVC), and arrhythmogenic left ventricular phenotype (ALVC).

In this narrative review, we aim to provide a comprehensive overview of the current state of knowledge on cardiomyopathies, offering a structured perspective on the topic, highlighting key updates, identifying gaps in the literature, and suggesting directions for future research (Table 1).

## 2. Imaging Modalities in Cardiomyopathies

### 2.1. Echocardiography: The First Line of Diagnosis

Echocardiography remains the cornerstone of cardiomyopathy diagnostics, primarily due to its wide availability, safety, and cost-effectiveness [4,5,6]. It is the first-line imaging modality recommended in the initial steps of the diagnostic pathway by the 2024 guidelines for assessing ventricular structure, wall thickness, and functional abnormalities in heart diseases, including cardiomyopathies [2]. Transthoracic echocardiography (TTE) can accurately assess left ventricular (LV) and right ventricular (RV) function, wall thickness, and chamber size, which are critical in diagnosing hypertrophic cardiomyopathy, dilated cardiomyopathy, and restrictive cardiomyopathy [4].

**Figure 1:**
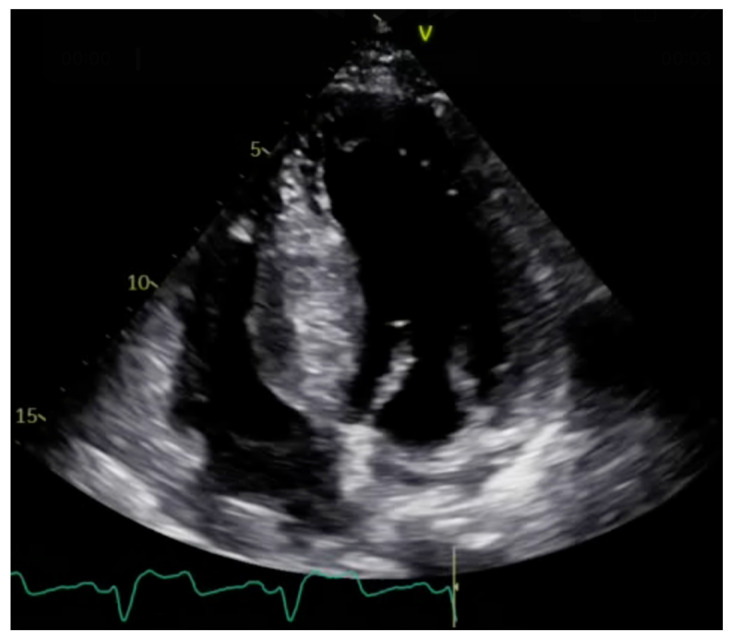
Transthoracic echocardiogram in patient with hypertrophic cardiomyopathy. Asymmetric septal hypertrophy.

**Figure 2:**
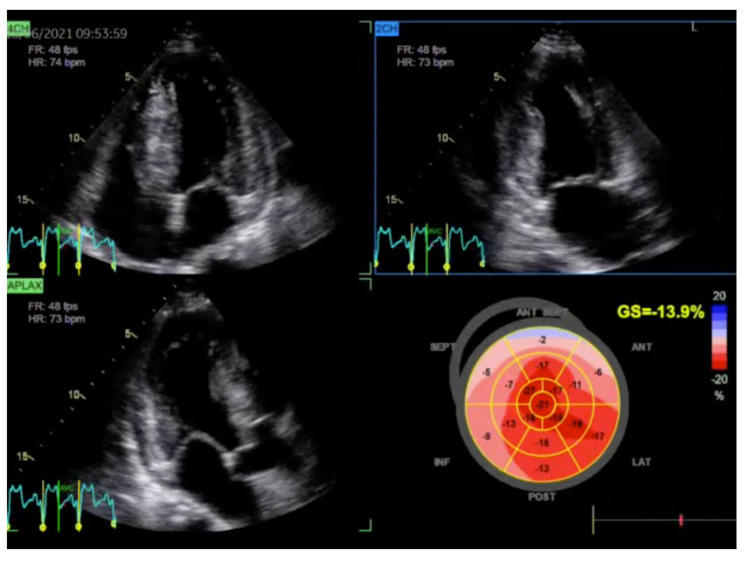
Longitudinal left ventricular strain assessment in patient with hypertrophic cardiomyopathy. Two-dimensional transthoracic echocardiogram. Moderately reduced strain (−13.9%).

One of the most critical features of HCM is the detection of asymmetric left ventricular hypertrophy (LVH), which is often localized to the baseline part of interventricular septum. A peak LV outflow tract (LVOT) gradient ≥30 mmHg defines obstructive HCM (Figure 1). TTE enables dynamic assessments of this gradient, particularly during Valsalva maneuvers or exercise, which helps reveal latent obstruction that may not be apparent at rest. Exercise stress echocardiography is recommended for symptomatic patients with LVOTO <50 mmHg at rest or during bedside maneuvers [7]. Moreover, advanced echocardiographic techniques, such as speckle tracking strain imaging, provide insights into myocardial mechanics, revealing subclinical myocardial dysfunction even in patients with preserved ejection fraction (Figure 2) [7,8,9].

**Figure 3:**
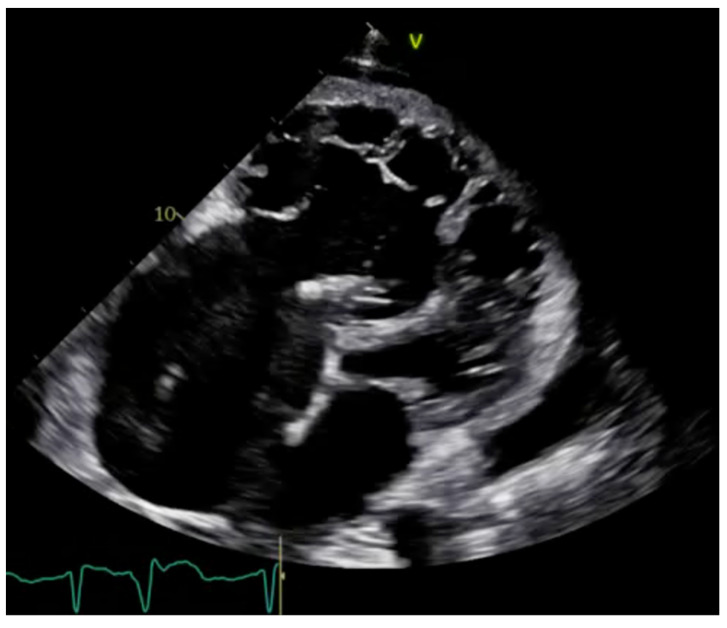
Arrhythmogenic right ventricular cardiomyopathy. Two-dimensional Transthoracic echocardiogram.

**Figure 4:**
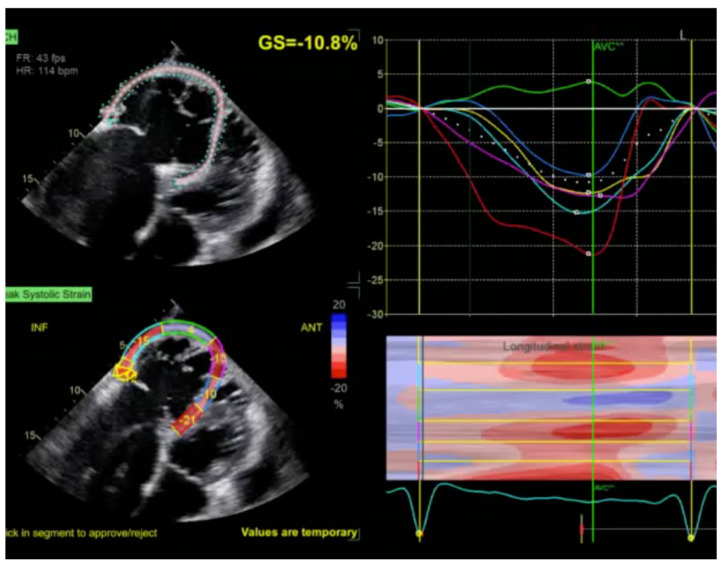
Longitudinal left ventricular strain assessment in patient with arrhythmogenic right ventricular cardiomyopathy. Very low right ventricular wall longitudinal strain (−10.8%).

For patients with suspected arrhythmogenic right ventricular cardiomyopathy (ARVC), echocardiography can identify global RV dysfunction, and regional RV akinesia, dyskinesia, and aneurysms [3]. Tissue Doppler imaging (TDI) of the lateral part of the tricuspid anulus and speckle tracking longitudinal strain are also very useful in the presentation of RV systolic dysfunction (Figure 3 and Figure 4). In biventricular and left ventricular arrhythmogenic cardiomyopathy, the morpho-functional abnormalities and structural alterations may include also left ventricle wall [3].

Hypertrophic cardiomyopathies, whether sarcomeric or non-sarcomeric, exhibit a variety of echocardiographic left ventricular hypertrophy (LVH) patterns. However, all strain components are reduced in these patients. Global longitudinal strain (GLS) provides an accurate estimation of LV function and may help in identifying the fibrosis, being the lowest regional strain values usually seen in hypertrophic and fibrotic hearts. These findings correlate with the cardiac magnetic resonance imaging results, a method that is considered the gold standard for evaluating wall thickness and regional fibrosis, thereby establishing speckle tracking automated functional imaging as a valuable marker of myocardial fibrosis [10]. Further, in the assessment of the pathophysiology of hemodynamic changes in HCM, it is useful to use the echocardiographic method of pressure–strain loop-derived global myocardial work index (GWI), which shows a significant deterioration of GWI in both diseases, even in patients with preserved left ventricular ejection fraction (LVEF) [11]. A much greater reduction in the GWI value in amyloidosis than in sarcomeric HCM may indicate a greater usefulness of the GWI index than the GLS assessment in the differential diagnosis of cardiac hypertrophy [12]. The assessment of left atrial strain has a similar value in differential diagnosis. The values of LA-reservoir, and conduit and contraction strain were found to be significantly worse in Monte IP studies in patients with amyloidosis compared to patients with sarcomeric HCM [13].

### 2.2. Cardiac Magnetic Resonance Imaging: Gold Standard

**Figure 5:**
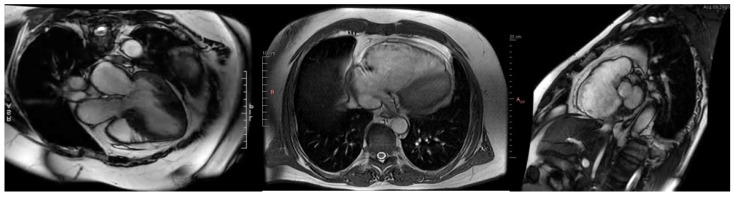
**Left**: Hypertrophic cardiomyopathy with asymmetric septal hypertrophy in cardiac magnetic resonance. Horizontal view. **Middle**: Arrhythmogenic right ventricular cardiomyopathy in magnetic resonance. Horizontal view. **Right**: Arrhythmogenic right ventricular cardiomyopathy in cardiac magnetic resonance Sagittal view.

Over the past decade, cardiac magnetic resonance has emerged as the gold standard for the detailed morphological and functional assessment of cardiomyopathies. CMR provides high-resolution imaging of the myocardium and is not limited by poor acoustic windows, which can sometimes compromise echocardiography. CMR should be performed at the beginning of the diagnostic process in any cardiomyopathy phenotype (Figure 5) [1,14]. This diagnostic modality is invaluable in differentiating HCM from phenocopies characterized by increased heart wall thickness (i.e., HCM mimics) or allowing for the determination of the proportions between the volume of intracellular and extracellular space, as it is the case in amyloidosis, where, unlike HCM, the extracellular space is significantly increased due to extracellular accumulation of amyloid [15,16,17].

One of CMR’s most valuable features is its ability to assess myocardial fibrosis using late gadolinium enhancement (LGE), which has both diagnostic and prognostic significance across all types of cardiomyopathies. LGE is a hallmark of myocardial fibrosis, which may occur due to ischemic or non-ischemic causes. In hypertrophic cardiomyopathy, the extent of LGE correlates with the degree of myocardial fibrosis and has been associated with an increased risk of sudden cardiac death (SCD) [2]. Therefore, the presence of significant LGE in patients with HCM might prompt discussions regarding the need for implantable cardioverter-defibrillator (ICD) placement, even in those without other major risk factors. Moreover, in ARVC, CMR is useful in detecting fatty or fibrofatty infiltration of the RV myocardium, which is a characteristic feature of the disease. CMR’s ability to identify even subtle abnormalities in RV function and structure has made it indispensable in the early diagnosis of ARVC, which is essential to prevent life-threatening arrhythmias (Figure 5, middle and right panel) [18,19]. In dilated cardiomyopathy, CMR can differentiate between ischemic and non-ischemic causes of LV dilation by detecting patterns of LGE consistent with myocardial infarction (transmural or subendocardial fibrosis) versus non-ischemic fibrosis (patchy midwall or subepicardial fibrosis). This distinction is critical for guiding appropriate therapy and determining prognosis [20,21,22]. CMR is essential in the diagnosis of a new cardiomyopathy phenotype, non-dilated left ventricular cardiomyopathy (NDLVC). The diagnosis requires the demonstration of non-ischemic LV scar or lipid infiltration, regardless of the presence of global or local dysfunction or the presence of generalized LV hypokinesis without the presence of scar [1]. For ARVC, CMR is the diagnostic imaging modality of choice due to its ability to visualize RV wall abnormalities, including fibrofatty infiltration and regional wall motion abnormalities, which are hallmarks of the disease. These features are often difficult to detect on echocardiography, particularly in the early stages of the disease [15,16,17,18]. CMR is helpful in the diagnosis of restrictive cardiomyopathy, especially in differentiating from the constrictive pericarditis in the presence of pericardial fibrosis in the absence of calcifications, which makes the diagnosis difficult using CT alone [23]. Overall, CMR can accurately quantify the extent and distribution of hypertrophy, assess the presence and severity of myocardial fibrosis, and identify hypertrophic phenotypes. The recent clinical implementation of T1 and T2 mapping has enhanced diagnostic precision. Quantitative mapping analysis holds potential as a surrogate marker for therapy efficacy in CA and AF diseases. Nevertheless, large-scale trials are required before mapping can be utilized as a follow-up tool to guide treatment. Currently, the presence and extent of LGE serve as additional independent prognostic factors in the stratification of sarcomeric HCM [24].

### 2.3. Computed Tomography: A Complementary Modality

**Figure 6:**
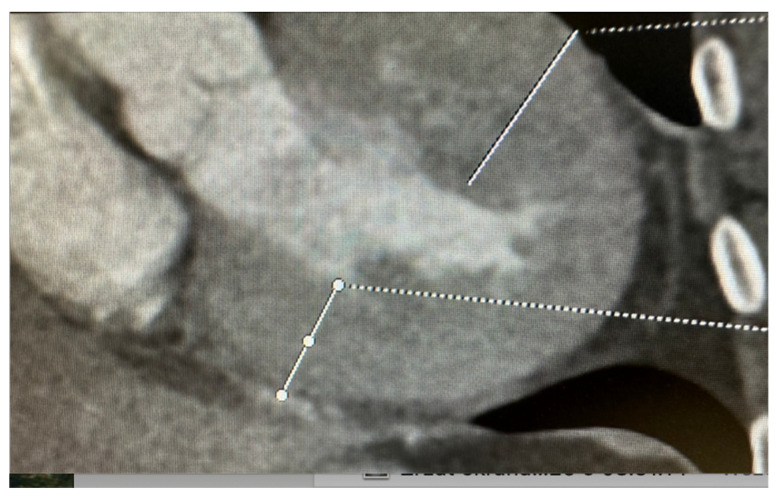
Cardiac tomography. Hypertrophic cardiomyopathy with large concentric left ventricular hyperthrophy.

While echocardiography and CMR are the primary imaging modalities for cardiomyopathies, computed tomography has a complementary role, particularly in the evaluation of coronary artery disease (CAD) in patients with suspected ischemic cardiomyopathy to rule out CAD or to exclude congenital malformation in coronary arteries. However, the role of CT in cardiomyopathies extends beyond coronary imaging [25,26]. In HCM, it very well illustrates the topography of myocardial hypertrophy (Figure 6) and allows measurement of left ventricular volume and ejection fraction. In restrictive cardiomyopathy, where pericardial diseases such as constrictive pericarditis may mimic restrictive physiology, CT can provide detailed images of the pericardium, allowing clinicians to distinguish between pericardial and myocardial causes of restrictive physiology [23]. CT also offers an alternative for patients with HCM who cannot undergo CMR due to the claustrophobia or possibility of imaging artifacts in patients with a pacemaker or ICD.

The advent of Photon-Counting CT (PCCT) has opened new avenues in cardiovascular imaging. This technique offers high-resolution images with rapid acquisition times and enables three-dimensional image reconstruction. Additionally, PCCT shows promise in the stratification of cardiovascular risk, potentially enhancing diagnostic accuracy and patient management [27,28].

### 2.4. Nuclear Imaging: Important in Selected Cases

The 99mTc-DPD/PYP/HDMP scintigraphy with SPECT is important in the differential diagnosis of cardiac hypertrophy when transthyretin cardiac amyloidosis is suspected [29]. While 18FDG-PET has no significant use in diagnosing the etiology of the heart pathologies with suspected cardiomyopathy, it can be useful in suspected cardiac sarcoidosis [30].

## 3. Genetic Foundations of Cardiomyopathies

Advances in the genetic testing technologies have significantly improved the ability to identify disease-causing variants in cardiomyopathies. Next Generation Sequencing (NGS), also known as high-throughput sequencing, has now become a routine testing method, allowing for extensive screening of genetic variants. Individuals typically have around 100 de novo variants and thousands of other rare single-nucleotide variants (SNVs). NGS is highly sensitive to identifying SNVs and small insertions or deletions. Pathogenic changes are most commonly found in the coding regions of genes, though deep-intronic variants have also been described in some HCM and DCM cases [30].

For both clinical and diagnostic purposes, gene and variant classifications are essential for standardizing the interpretation and reporting of genetic findings. Genes are classified, based on the strength of evidence linking them to the disease, into categories such as definitive, strong, moderate, limited, disputed, or no known disease relationship (Table 2) [30]. Many genes linked to monogenic cardiomyopathies have been evaluated, and only a small subset has sufficient evidence supporting their association with specific phenotypes. Genes with limited or disputed associations are considered genes of uncertain significance (GUS), as per ClinGen recommendations [31].

Variant classification follows the guidelines established by the American College of Medical Genetics and Genomics (ACMG) in 2015. The traditional terms “mutation” and “polymorphism” have been replaced by “variant,” with variants classified into five categories: pathogenic (P), likely pathogenic (LP), variants of uncertain significance (VUS), likely benign (LB), and benign (B). This classification applies to variants in Mendelian genes with clear links to clinical phenotypes, and only pathogenic and likely pathogenic variants are considered clinically significant [36,37,38]. Criteria for classifying variants take into account information from genetic databases, bioinformatic analyses, functional studies, and clinical data, including family segregation studies and de novo occurrences. This emphasizes the importance of collaboration between clinicians and laboratories for accurate genetic testing.

## 4. Genetic Testing Methods, Gene and Variant Classification

Advances in the genetic testing technologies have significantly improved the ability to identify disease-causing variants in cardiomyopathies. Next Generation Sequencing (NGS), also known as high-throughput sequencing, has now become a routine testing method, allowing for extensive screening of genetic variants. Individuals typically have around 100 de novo variants and thousands of other rare single-nucleotide variants (SNVs) [28]. NGS is highly sensitive to identifying SNVs and small insertions or deletions. However, detecting copy number variants (CNVs) is more challenging and requires specific bioinformatics analysis. Techniques like Multiplex Ligation Dependent Probe Amplification (MLPA) and Array Comparative Genome Hybridization (aCGH) are still considered the gold standards for identifying CNVs, which affect approximately 0.6% of DCM patients, 0.35–1.5% of HCM patients, and 1% of ARVC patients [39]. Pathogenic changes are most commonly found in the coding regions of genes, though deep-intronic variants have also been described in some HCM and DCM cases [40,41,42]. In some instances, mitochondrial variants are responsible for cardiomyopathies, especially in severe pediatric cases with additional non-cardiac symptoms. Such mitochondrial disorders can be difficult to distinguish clinically from other genetic cardiomyopathies.

Guidelines for variant interpretation have become more gene- and phenotype-specific, such as for variants in the MYH7 or MYBPC3 genes in HCM, DCM, and RCM patients. Reclassification of variants is sometimes necessary as new data emerge, with the majority of reclassifications downgrading the pathogenicity of a variant. Only a small percentage (0.24%) of VUS have been upgraded to higher pathogenicity in large genetic databases [43].

## 5. Genetic Testing and Imaging in Risk Stratification

The risk of sudden cardiac death is a primary concern in cardiomyopathies, particularly in HCM, DCM, and ARVC, where ventricular arrhythmias can lead to fatal outcomes. Imaging plays a pivotal role in SCD risk stratification, guiding the decision to implant a cardioverter-defibrillator. Currently, there is no single risk assessment algorithm for all cardiomyopathy phenotypes, although it should be noted that several risk factors are common to each phenotype. These include indicators assessed using imaging methods.

### 5.1. Hypertrophic Cardiomyopathy

HCM is typically caused by mutations in genes encoding sarcomeric proteins and is inherited in an autosomal dominant manner. X-linked forms include Anderson–Fabry disease (GLA gene) and Danon disease (LAMP2 gene), while mitochondrial or recessive inheritance, such as in the ALPK3 gene, is often linked to syndromic or pediatric cases. The key genes associated with HCM include those with a definitive connection to isolated HCM, such as MYBPC3, MYH7, TNNT2, TNNI3, TPM1, ACTC1, MYL2, and MYL3. There are also genes with moderate evidence for isolated HCM, including CSRP3, JPH2, TNNC1, and TRIM63. Some genes are associated with syndromic HCM, where HCM appears as part of a broader condition. These include genes involved in RASopathies, as well as genes like ABCC9, BAG3, CAV3, COX15, CRYAB, FXN, GAA, LDB3, and SLC25A4, which contribute to HCM along with other symptoms. Certain genes such as DES, FHL1, FLNC, GLA, LAMP2, PRAKG2, PTPN11, RAF1, RIT1, and TTR may cause HCM as the sole symptom. Finally, genes like ACTN2 and PLN contribute to mixed phenotypes, displaying characteristics of both HCM and other cardiomyopathies.

Most variants that cause HCM are single-nucleotide variants (SNVs) found in the coding regions of genes, but deep-intronic variants and copy number variants (CNVs) are also reported, though less frequently [44]. Genetic testing detects pathogenic variants in about 30–65% of HCM patients, with MYH7 and MYBPC3 being the most common. Genetic results can confirm diagnosis, guide cascade testing in family members (potentially exempting those without the variant from further monitoring), and facilitate targeted therapies like recombinant alpha-galactosidase in Fabry disease or tafamidis in ATTR amyloidosis.

The risk of death from cardiovascular causes in adults with HCM is estimated to be approximately 1–2% per year, including heart failure, thromboembolic complications, and SCD [45]. The risk of SCD in adults is relatively low; however, it increases in children over the age of 6 years with the peak of annual SCD risk being about 1.2–1.5 between 9 and 15 years old [46,47]. The decision to implant an ICD for primary prevention is complex. There are several major risk factors for SCD, which are further used to calculate the 5-year risk and widely used for shared decision-making before ICD implantation: family history of SCD, massive LVH, unexplained syncope, apical aneurysm, and LVEF < 50% [2]. Overall, the HCM-risk score includes age, maximum LV wall thickness, left atrial size, LVOT gradient, family history of SCD, non-sustained VT, LGE assessed by CMR, and unexplained syncope.

The updated 2024 guidelines emphasize the importance of LGE on CMR in stratifying SCD risk in patients with HCM, noting that the extent of LGE, comprising ≥15% of LV mass, correlates with the risk of ventricular arrhythmias [48,49]. In addition to LGE, other imaging parameters such as LV wall thickness, LV outflow tract gradient, and the presence of apical aneurysms on echocardiography are also considered when assessing SCD risk. In hypertrophic cardiomyopathy, the degree of left ventricular hypertrophy (LVH) is a significant predictor of sudden cardiac death. Imaging techniques, particularly echocardiography and cardiac magnetic resonance, are essential for quantifying wall thickness, which helps in risk stratification [50]. The updated 2024 guidelines highlight that patients with a maximum LV wall thickness of ≥30 mm are at increased risk for SCD and may benefit from the implantation of an ICD.

Apical aneurysms, often associated with advanced hypertrophic cardiomyopathy, are another imaging feature that increases the risk of sudden cardiac death. These aneurysms are best visualized using CT or CMR, as they are sometimes missed on echocardiography. Patients with apical aneurysms are at an increased risk for ventricular arrhythmias and thromboembolic events, and imaging is crucial for identifying those who may benefit from ICD implantation or anticoagulation [51,52]

### 5.2. Dilated Cardiomyopathy and Non-Dilated Left Ventricular Cardiomyopathy

DCM has a broad range of causes, including genetic and non-genetic factors. In clinical practice, approximately 15–35% of patients with DCM have their diagnosis confirmed by genetic testing [53]. Most genetic cases of DCM follow an autosomal dominant inheritance pattern, although recessive, X-linked, or mitochondrial inheritance can also occur. More than 200 genes have been associated with DCM, with about 60 of these genes validated. The most important genes linked to DCM include those with definitive evidence for isolated DCM, such as BAG3, DES, DSP, FLNC, LMNA, MYH7, PLN, RBM20, SCN5A, TNNC1, TNNT2, and TTN. In syndromic DCM, genes such as DMD (associated with Becker/Duchenne muscular dystrophy) are key contributors. Additionally, genes with moderate evidence for isolated DCM include ACTC1, ACTN2, JPH2, NEXN, TNNI3, TPM1, and VCL. Among these, truncating variants in TTN (TTNtv) are particularly common, present in more than 10% of DCM patients, and account for 40–50% of positive genetic results. Other frequently identified variants include those in BAG3, DSP, LMNA, MYH7, TNNT2, FLNC, and RBM20. Most pathogenic variants in DCM are single-nucleotide variants (SNVs) found in coding regions, although deep-intronic or copy-number variants (CNVs) are identified in 0.5–1% of cases, most commonly affecting DMD, BAG3, and LMNA genes [1].

Non-dilated left ventricular cardiomyopathy (NDLVC) is generally caused by mutations in genes like DSP, FLNC, DES, LMNA, and PLN, and is inherited in an autosomal dominant manner. Genetic testing is recommended for all patients diagnosed with NDLVC. As this is a new classification, the percentage of positive genetic results in this group is not yet known. This category includes patients previously classified under conditions like DCM without dilation, arrhythmogenic DCM, or ARVC with left ventricular involvement. Genetic results have prognostic value and may inform decisions regarding ICD implantation, as well as enabling cascade testing in relatives. As with DCM, high-intensity sports are not recommended for patients with pathogenic variants in LMNA or TMEM43 [1].

In DCM the primary imaging findings include left ventricular dilatation, reduced systolic function, and fibrosis. Echocardiography remains the first-line diagnostic tool but CMR provides a more detailed evaluation. CMR is a reference method in LVEF assessment. Echocardiography tends to underestimate the LVEF; therefore, performing a CMR testing may reclassify the indications for ICD implantation in up to 35% of patients [54].

According to the current ESC 2022 recommendations, ICD implantation should be considered in patients with DCM/HNDCM (hypokinetic/non-dilated cardiomyopathy), symptomatic heart failure (NYHA class II–III), and LVEF ≤ 35% after ≥3 months of OMT [55]. However, EF shows poor power in identifying patients with DCM at risk for SCD. It is advisable to additionally take into account other factors, including genetic variants associated with high arrhythmic risk (e.g., LMNA, EMD, DSP, PKP2), as well as a history of syncope and the presence of LGE on CMR [56,57,58].

### 5.3. Arrhythmogenic Right Ventricular Cardiomyopathy

ARVC is primarily caused by mutations in genes encoding desmosomal proteins, which are involved in cell-to-cell adhesion. Familial ARVC is usually inherited in an autosomal dominant pattern, with incomplete penetrance influenced by age, sex, and physical activity. The first ARVC-causing variant was identified in the JUP gene in 2000, in a recessive form known as Naxos syndrome. Genes with definitive links to ARVC, according to ClinGen, include DSC2, DSG2, DSP, JUP, PKP2, and TMEM43, while genes with moderate evidence include DES and PLN. The role of FLNC in ARVC is currently under expert review. Genetic testing for ARVC should include the genes PKP2, DSP, DSC2, DSG2, JUP, TMEM43, PLN, FLNC, DES, and LMNA. Approximately 60% of ARVC patients receive a positive genetic test result, with PKP2, DSP, DSC2, and DSG2 being the most commonly affected genes. Since 1–4% of ARVC patients have CNV changes, testing for these should be included in the genetic analysis.

ARVC is a rare form of cardiomyopathy, yet it poses a significant proarrhythmic potential that can lead to SCD, an event often occurring in young athletes during physical activity. The risk stratification algorithm for determining ICD implantation for primary prevention is predicated on relatively small observational cohorts and expert consensus [59,60,61,62]. As cited in the ESC 2023 recommendations [1], the 2019 Heart Rhythm Society (HRS) expert consensus statement presents a risk assessment model predicated on factors such as gender, age, recent syncope, nsVT, VT count, and number of leads with T-wave inversion (TWI), as well as Right Ventricular Ejection Fraction (RVEF) < 40%, LVEF < 45%, and Sustained Monomorphic Ventricular Tachycardia (SMVT) at Programmed Electrical Stimulation (PES) [63]. For the assessment of RVEF and LVEF, it is recommended to employ 3D echocardiography or cardiac magnetic resonance.

### 5.4. Restrictive Cardiomyopathy

RCM, the least common form of cardiomyopathy, is characterized by restrictive left ventricular pathophysiology, such as a rapid rise in ventricular with only minor increases in filling volume, a result of increased myocardial stiffness. The hallmark of RCM is the persistent presence of restrictive pathophysiology, diastolic dysfunction, non-dilated ventricles, and atrial dilatation, irrespective of ventricular wall thickness and systolic function [64]. RCM is caused by variants in sarcomeric, cytoskeletal, or Z-line proteins, including TNNI3, TNNT2, ACTC1, MYH7, MYBPC3, DES, BAG3, FLNC, TTN, TPM1, MYPN, MYL3, and MYL2. Familial RCM is generally inherited in an autosomal dominant manner, with de novo variants often observed. The same genetic variant can lead to RCM, HCM, or a mixed phenotype in different members of the same family. The first family-related RCM-causing variants were identified in TNNI3 in 2003, with BAG3 mutations discovered in 2018 [65]. RCM can also result from transthyretin amyloidosis, caused by pathogenic variants in the TTR gene. Genetic testing is especially important for diagnosing syndromic RCM, such as Anderson–Fabry disease (GLA), glycogen storage diseases (PRAKG2), Danon disease (LAMP2), and amyloidosis (TTR), as it allows for cascade testing of family members. This phenotype is correlated with a poorer prognosis compared to other cardiomyopathy phenotypes. The risk factors for mortality include restrictive physiology as assessed by echocardiography, diminished LV systolic function, left atrial enlargement, syncope, and signs of ischemia. Currently, there is no recommended algorithm delineating the pathway for ICD implantation in primary prevention [1].

## 6. Conclusions and Future Directions

Genetic testing plays a critical role in diagnosing and managing various forms of cardiomyopathies, including hypertrophic, dilated, restrictive, and arrhythmogenic cardiomyopathies. Identification of pathogenic variants helps confirm diagnoses, informs risk assessment for sudden cardiac death, and guides family screening through cascade testing. Both American and European cardiology societies place genetic counseling and testing for cardiomyopathies as class I of recommendations. Early genetic testing not only aids in treatment decisions, including the use of targeted therapies, but also helps predict disease progression and guides lifestyle recommendations, such as avoiding high-intensity sports for at-risk individuals.

Cardiac imaging continues to play a vital role in the diagnosis, phenotyping, risk stratification, and management of cardiomyopathies. The latest ESC and AHA/ACC guidelines reflect the growing importance of advanced imaging techniques, including echocardiography, cardiac magnetic resonance, and computed tomography, in guiding therapeutic decisions and improving patient outcomes. Future advancements, including artificial intelligence, strain imaging, and molecular imaging, are poised to revolutionize the field, offering more precise diagnostic tools and personalized approaches to treatment. AI approaches have been applied to cardiomyopathies by using ECG, genetic data, and cardiac imaging modalities such as echocardiography, CMR, and nuclear imaging [24,66].

AI is currently utilized in echocardiography for automated chamber quantification, ejection fraction calculation, strain measurement, and other LV functional assessments, while speckle tracking technology and myocardial movement can be visualized and ventricular volumes can be automatically measured [67]. AI has been used to detect HCM and cardiac amyloidosis in an automated fashion quickly and accurately. Potentially, AI can complement the field of cardiovascular genomics by assessing the quality of genetic samples obtained (e.g., DNA, RNA, exome), improving informatics pipelines for variant calling, interpreting raw data in the context of clinical guidelines, transforming genetic files, predicting variant pathogenicity, mapping an individual’s sequence to genome references, and identifying any clinically actionable mutations [68]. AI applications in CMR have contributed significantly to the acceleration of image acquisition and analysis in all aspects of LV and right ventricle volumetry and mass measurements [69,70].

In genetic testing, AI can complement the field of cardiovascular genomics by assessing the quality of genetic samples obtained (e.g., DNA, RNA, exome), improving informatics pipelines for variant calling, interpreting raw data in the context of clinical guidelines, transforming genetic files, predicting variant pathogenicity, mapping an individual’s sequence to genome references, and identifying any clinically actionable mutations [68].

Although, while the development and application of AI in cardiomyopathies offer great promise, there are potential challenges to overcome. Because most cardiomyopathies are relatively rare, the sample size of datasets is typically smaller than other AI datasets. This makes it difficult to develop risk prediction models, especially DL-based ones, as they benefit significantly from using large datasets. Even in the setting of a perfect algorithm, if the data being imputed is of poor quality or biased or missing, the interpretation and application in a pragmatic clinical setting will be limited. Especially in the field of echocardiography, the contribution and dexterity of the clinician are essential to allow a good analysis by any algorithm. Given that models are often derived from high-quality databases with meticulously obtained ECG and cardiac images in well-phenotyped patients in large medical centers, their application in routine clinical practice and in real-world settings might not be reproducible.

Genetic and biomarker integration into imaging protocols is another promising direction. As understanding of the genetic underpinnings of cardiomyopathies grows, there is an opportunity to combine genetic data with advanced imaging modalities to better predict disease progression and tailor treatment plans. These emerging technologies hold promise to enhance the ability to diagnose the disease early, predict adverse outcomes, and tailor interventions to individual patient needs. This highlights the need for ongoing collaboration between clinicians, data scientists, and policymakers to realize the full potential of AI in cardiovascular imaging and genetics while ensuring ethical and data-driven implementation. As the field continues to evolve, addressing these challenges will be crucial for potentially revolutionizing diagnostic capabilities and improving patient outcomes.

## Figures and Tables

**Table 1:** Key points summary.

Section	Key Points
Overview	Cardiomyopathies represent a high unmet medical need and global burden, being a frequent cause of heart failure or sudden death. Integrated phenotyping and genetic testing are crucial for timely diagnosis, enabling patients to be eligible for preventive measures and emerging therapies.
Imaging Modalities	Echocardiography: first-line tool; assesses LV/RV structure, strain imaging for early dysfunction. CMR: gold standard for fibrosis (LGE), morphology, and phenotyping. CT: evaluates coronary anomalies; Photon-counting CT for emerging applications. Nuclear Imaging: differentiates amyloidosis; limited role in other types.
Genetic Testing	NGS identifies pathogenic variants (e.g., MYBPC3, MYH7, TTN, LMNA). Cascade testing guides familial screening and therapy.
Risk Stratification	Key factors: LV wall thickness, LGE extent, apical aneurysm, genetic variants. ICD decisions based on combined imaging and genetic insights.
Future Directions	AI enhances imaging (strain, fibrosis quantification) and genetic integration. Emerging tools: molecular imaging, Photon-Counting CT.

**Table 2:** Overview of the most common genes, with definite/strong levels of evidence, associated with monogenic, non-syndromic cardiomyopathic phenotypes. Adapted based on the 2023 ESC Guidelines for the management of cardiomyopathies [1,32,33,34,35].

Phenotype	Associated Genes	Inheritance Pattern	Additional Features
HCM	Sarcomeric: MYBPC3, MYH7, TNNT2, TNNI3, TPM1, ACTC1, MYL2, MYL3; Non-Sarcomeric: GLA, LAMP2, ALPK3;	Autosomal Dominant, X-Linked, Mitochondrial, Recessive	Syndromic or Pediatric Cases
DCM	BAG3, DSP, FLNC, LMNA, MYH7, RBM20, TNNT2, TTN	Varies	-
ARVC	DSC2, DSG2, PKP2	Varies	-

ACTC1: Actin Alpha Cardiac Muscle 1; ALPK3: Alpha Kinase 3; BAG3: BCL2 Associated Athanogene 3; CAV3: Caveolin 3; COX15: COX15 Cytochrome C Oxidase Assembly Factor; CRYAB: Crystallin Alpha B; DSC2: Desmocollin 2; DSG2: Desmoglein 2; DSP: Desmoplakin; FLNC: Filamin C; FXN: Frataxin; GAA: Glucosidase Alpha Acid; GLA: Galactosidase Alpha; LAMP2: Lysosomal Associated Membrane Protein 2; LDB3: LIM Domain Binding 3; LMNA: Lamin A/C; MYBPC3: Myosin Binding Protein C3; MYH7: Myosin Heavy Chain 7; MYL2: Myosin Light Chain 2; MYL3: Myosin Light Chain 3; PLN: Phospholamban; PRAKG2: Protein Kinase cGMP-Dependent Type II; PTPN11: Protein Tyrosine Phosphatase Non-Receptor Type 11; RAF1: RAF1 Proto-Oncogene, Serine/Threonine Kinase; RBM20: RNA Binding Motif Protein 20; RIT1: Ras Like Without CAAX 1; SLC25A4: Solute Carrier Family 25 Member 4; TNNC1: Troponin C1, Slow Skeletal and Cardiac Type; TNNI3: Troponin I3, Cardiac Type; TNNT2: Troponin T2, Cardiac Type; TPM1: Tropomyosin 1; TTN: Titin.
